# An open-label randomized multi-Centre study to evaluate anterior controllable Antedisplacement and fusion versus posterior Laminoplasty in patients with cervical ossification of the posterior longitudinal ligament: study design and analysis plan (STAR)

**DOI:** 10.1186/s12891-021-04645-3

**Published:** 2021-09-08

**Authors:** Yu Chen, Jingchuan Sun, Dan Han, Xiaoqiu Yuan, Yuan Wang, Yongfei Guo, Xihua Zhong, Jiangang Shi

**Affiliations:** 1 Department of Orthopedic Surgery, Spine Centre, Changzheng Hospital, Naval Medical University, No. 415 Fengyang Road, Shanghai, 200003 People’s Republic of China; 2 Data Statistics Centre, Shanghai KNOWLANDS MedPharm Consulting Co., Ltd., No. 1839 Qixin Rd, Shanghai, 201101 People’s Republic of China

**Keywords:** Anterior controllable antedisplacement and fusion, Posterior laminoplasty, Clinical outcome, Cervical ossification of the posterior longitudinal ligament, Randomized controlled trial

## Abstract

**Background:**

In treating patients with cervical ossification of the posterior longitudinal ligament (COPLL), a novel surgery technique - anterior controllable antedisplacement and fusion (ACAF) suggested promising clinical benefits in recent exploratory studies.

**Methods:**

This is a multicentre, randomized, open-label, parallel-group, active controlled trial that will compare the clinical benefits of ACAF versus conventional posterior laminoplasty (LAMP) in severe COPLL patients. A total of 164 patients will be enrolled and randomized in a 1:1 ratio to either ACAF or LAMP group. The primary efficacy measure is cervical- Japanese Orthopaedic Association (C-JOA) recovery rate at 12 months post operation, which is to be derived by Hirabayashi’s method from JOA data (range, 0 [worst] to 17 [normal condition]). Other important secondary efficacy endpoints include visual analogue scale (VAS) pain score (range, 0 [no pain] to 10 [most severe]), 10-item neck disability index (NDI, a total range of 0 to 50 points, the highest index the worst) and 6-level Nurick disability grade (range, 0 [mild] to 5 [severe]). Safety endpoints including adverse events, perioperative complications, and adverse events of special interest will also be assessed in this study. Full analysis set for baseline and efficacy data analyses according to the intention-to-treat principle will be established as the primary analysis population. Analysis of covariance (ANCOVA) will be used to analyze the C-JOA recovery rate, with random stratification factors (if appropriate) and the treatment group as fixed factors, and the baseline level of C-JOA score as covariate.

**Discussion:**

This study is designed to demonstrate the clinical benefits of ACAF as compared to conventional LAMP in COPLL patients. It will provide clinical evidence that the novel surgery technique – ACAF might be more favorable in treating patients with severe cervical ossification of the posterior longitudinal ligament. (Words: 290).

**Trial registration:**

ClinicalTrials.gov number, NCT04968028.

## Background

Ossification of the posterior longitudinal ligament (OPLL) is a pathologic process of lamellar bone deposition at the site of the posterior longitudinal ligament (PLL) [1]. And it often occurs in the cervical spine, which was first noticed in the Japanese patients and reported more commonly in Asian countries than North America, with a prevalence rate of 2 to 4% in Japan [2], 3.1% in China [3], 5.1% in India [4] and 2.5% in USA [5], respectively. As one kind of chronic and multifactorial disease, it usually causes spinal cord compression over time and lead to a reduced range of cervical motion [1],, and further serious neural injury like axial neck pain, diminished sensation, motor weakness, and changes in balance or gait stability, or more severely, incontinence, paralysis, severe respiratory failure and even death [1, 4–6].

Non-operative management can be often undertaken for asymptomatic patients without severe cord compression or myelomalacia on radiographic scans [6–9]. However, for patients with neurologic symptoms such as myelopathy or radiculopathy or evidence of spinal cord compression, surgical intervention is required and also the only effective treatment options to date [6–10]. Mainstream surgical approaches available currently are those utilizing anterior (ACCF, anterior cervical corpectomy and fusion), posterior (LAMP, laminoplasty; LF, laminectomy and fusion), or combined anteroposterior strategy, each with different levels of risks and benefits [1, 11]. Anterior approaches are technically more demanding and associated with higher rates of spinal cord injury, cerebrospinal fluid leak, dysphagia, dysarhtria, hoarseness but offer direct decompression for ventral OPLL pathology. Although posterior own higher safety, associated with higher rates of C5 palsy, axial pain, and may allow for ossification progression that lead to recurrence of neurological deterioration [11, 12]. Even so, high level of clinical evidence is still inconclusive so far [12], therefore, surgeons and patients have to subjectively decide on surgery strategy after individualizing the potential benefits of surgical decompression against the potential medical and surgical complications [1, 12].

To make full use of the estimated advantages of anterior and posterior approaches and maximally avoid disadvantages, we have designed a novel surgery technique - anterior controllable antidisplacement and fusion (ACAF) strategy in a hope to achieve anterior direct decompression without removing the OPLL [13–15]. Our preliminary results in the recent exploratory studies [13, 14], along with those found in a Korean case series report (*N* = 24) [16] suggested that ACAF technique is an effective and safe option for anterior decompression surgery in severe OPLL patients. Given a worldwide paucity of level-I evidence comparing surgical approaches to OPLL [11] and potential expert consensus or clinical practice guideline to come out, we designed this adequate and well-controlled investigation across multiple study sites in China. Here we present its rationale, methodology and analysis plan on how to compare the clinical effectiveness of ACAF technique against LAMP among severe COPLL patients. The randomized controlled trial aims to provide clinical evidence that ACAF might be more favorable in treating severe COPLL patients.

## Methods and design

### Study design

**Fig. 1:**
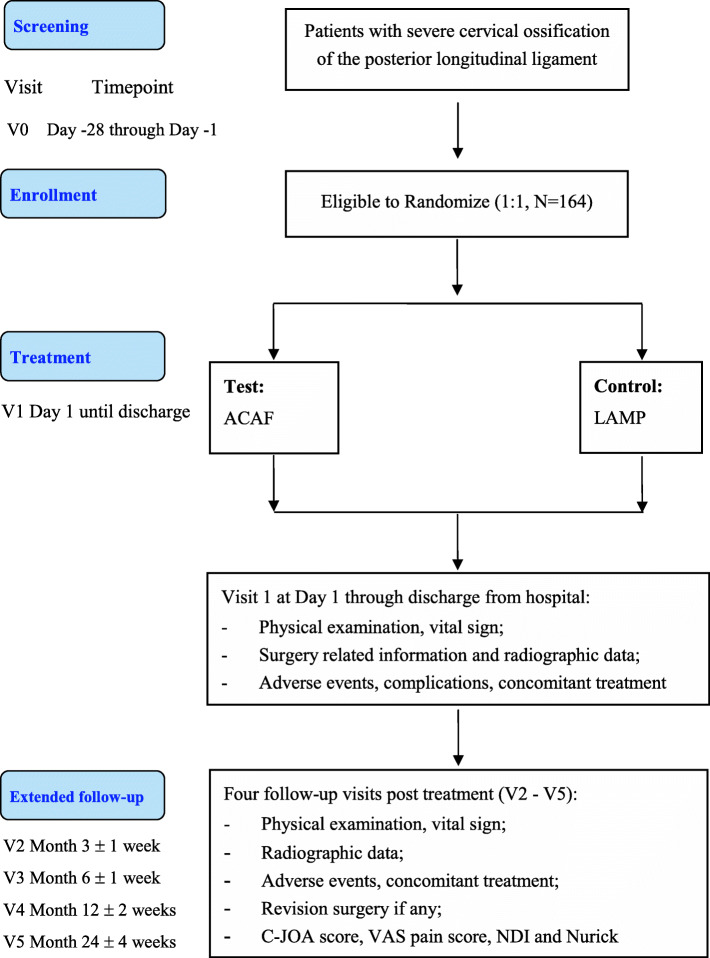
Study flow chart. Abbreviations: ACAF, anterior controllable antedisplacement and fusion; C-JOA, cervical Japanese Orthopaedic Association; LAMP, posterior laminoplasty; NDI, neck disability index; VAS, visual analogue scale

The STAR study (ClinicalTrials.gov number, NCT04968028) is a multicentre, randomized, open-label, parallel-group, active controlled trial that aims to demonstrate more favorable clinical benefits of ACAF as compared to conventional LAMP in severe COPLL patients. It will be conducted following the Consolidated Standards of Reporting Trials (CONSORT) statement (http://www.consort-statement.org/). Approximately twelve clinical centres in China will participate in this study. A brief flowchart of this study is provided in Fig. 1.

### Study patients

A total of 164 eligible patients will be enrolled and randomized after screening at the study sites. The following are the inclusion criteria:

1. The patient’s age is 18–70 years old, regardless of gender;
2. The patient was diagnosed as severe ossification of the posterior longitudinal ligament (X-ray or computed tomography [CT] showed ossification of the posterior longitudinal ligament). The imaging findings showed occupied ratio ≥ 60% or involved three or more segments.
3. Symptoms of spinal cord and nerve root compression, or accompanied by spinal cord compression symptoms such as dysfunction of urination and defecation, conservative treatment was ineffective or aggravated gradually;
4. The participant (or his legal guardian) can sign the informed consent.
  The exclusion criteria are as follows:
5. Congenital malformations (including but not limited to occipitocervical malformations, congenital cervical fusion, cervical related neurovascular malformations), ossification of cervical ligamentum flavum, cervical trauma, cervical cancer, cervical tuberculosis and other inflammatory diseases;
6. Patients with other spinal diseases such as thoracolumbar vertebrae that affect clinical symptoms; Patients with motor neuron diseases such as amyotrophic lateral sclerosis and other nervous system diseases;
7. The symptoms were aggravated due to recent trauma;
8. Patients who participated in other clinical trials in recent 3 months;
9. The participant (or his legal guardian) with mental illness and cognitive impairment cannot give full informed consent;
10. Those who are in poor health and cannot tolerate surgery; the patients were not suitable for surgical treatment after preoperative examination.

### Recruitment and randomization process

Before enrollment, there will be one pretreatment screening visit at study site office, during which each participant will be assigned a unique identification number.

Once considered eligible for entry, these COPLL patients will be randomly assigned to one of two study treatment groups, e.g. either ACAF or LAMP surgery in a 1:1 ratio. A stratified randomization using minimization technique [17, 18] will be carried out, stratified by study site, occupation ratio (< 60% versus ≥60%), K-line status (negative versus positive) and count of vertebrae involved (< 3 versus ≥3). Random assignment is generated by an independent statistician and implemented via central randomization mobile phone APP (Shanghai KNOWLANDS MedPharm Consulting Co., Ltd.). In order to avoid potential selection bias, the randomization sequence is concealed in APP from both clinical staff and patients until assignment. Hence, neither any investigators nor patients can influence which treatment group the study patients are assigned to.

### Description of the interventions

The enrolled subjects will be randomized to undergo the novel ACAF or conventional LAMP surgery. In order to minimize the potential bias of the trial data, all patients would be operated on by senior spine surgeons from spine surgery team at each site who are highly experienced in performing the two trial interventions. All patients in the study will accept the same surgical devices. All the consumables used in the operation are from the same manufacturer, and the operations will be carried out in strict accordance with the unified standard.

#### ACAF group

**Fig. 2:**
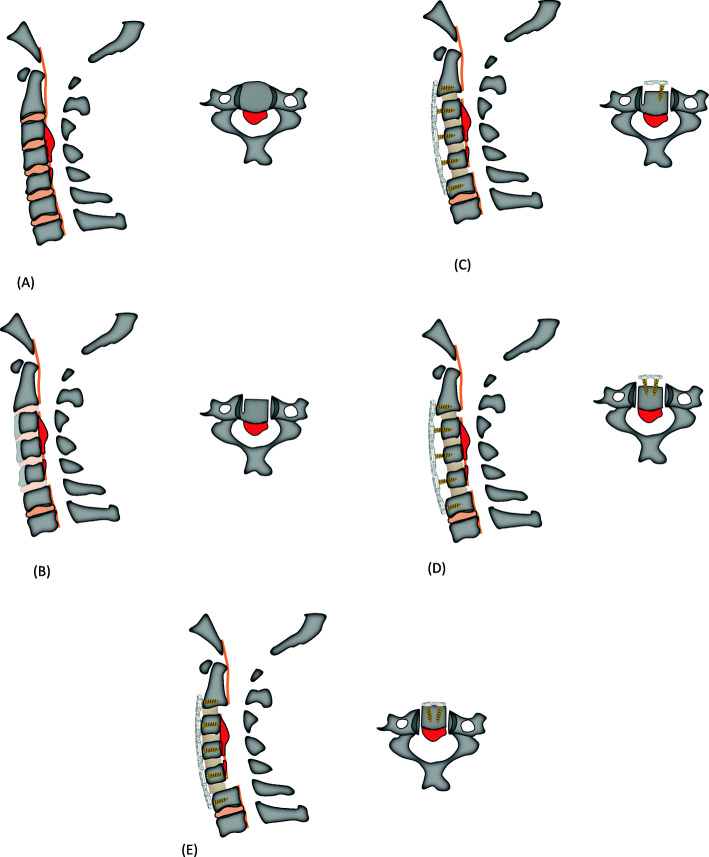
Schematic diagram of ACAF technique with steps A through E: (**A**) Expose and confirm surgical levels; (**B**) Remove discs, decompress at the levels cephalic and caudal to OPLL and remove the anterior portion of the middle vertebral bodies with OPLL; (**C**) Place interbody cages and create a groove on the left side, and fix anterior plate; (**D**) Create a groove on the right side, and isolate the vertebrae with OPLL and (**E**) Evaluate the vertebrae with OPLL. Sagittal (left) and transverse (right) sectional images of the cervical spine. Abbreviations: ACAF, anterior controllable antedisplacement and fusion; OPLL, ossification of the posterior longitudinal ligament

The surgical steps of ACAF were as follows: (1) a standard right-side Smith–Robinson approach was performed to expose the cervical spine, and intraoperative fluoroscopy was used to confirmed surgical levels. (2) The involved disc tissue was completely removed. The posterior longitudinal ligament was only cut down at the levels cephalic and caudal to OPLL, and the osteophytes at the anterior and posterior borders of vertebrae were appropriately removed for placing cages at the middle disc levels. (3) The anterior portion of the middle vertebral bodies with OPLL was appropriately removed according to the thickness of ossification. Suitable cages filled with autologous bone fragments were placed into each intervertebral space. (4) On the left side of the vertebra, high-speed burr and Kerrison rongeur were used to create a 2-mm-wide groove on the lateral side of vertebral body, approximately at the medial border of the transverse foramina. After that, an appropriate curved plated was fixed to the caudal and cephal ad vertebrae. On the middle vertebrae, screws were inserted halfway for temporary fixation. (5) On the right side of the vertebrae, as had performed on the left side, a similar groove was also created, and the vertebrae with OPLL were completely isolated. (6) Finally, the loose screws on the middle vertebrae were tightened to achieve a gradual evaluation of the vertebrae with OPLL, resulting in decompression of the spinal cord (Figs. 2A – 2E). The SKYLINE anterior cervical plate system with PEEK cage (Depuy Synthes Spine, Inc., Raynham, MA) was used in all patients. After surgery, all patients were immobilized in a Philadelphia collar for 6 to 8 weeks.

#### LAMP group

The surgical procedure of laminoplasty will be done as described in the literature using the ARCH miniplate fixation system (Depuy Synthes Spine, Inc., Raynham, MA). After surgery, all patients will be immobilized in a Philadelphia collar for 3 to 4 weeks.

As for the other treatments other than study surgery, including the perioperative treatment, study patients in the two groups both will follow Chinese Experts Consensus (2018) [19].

### Study visits

Six study visits per subject are scheduled in the study as follows: pre-treatment visit (Day − 28 to Day − 1), perioperative visit (Day 1 until discharge) and follow-up visit at month 3 ± 1 week, month 6 ± 1 week, month 12 ± 2 week and month 24 ± 4 week (Month 3, 6, 12 and 24 post treatment). These visits will be made at study site office or patient ward as appropriate. At scheduled visits, data relating to demography, vital sign, operating time, estimated blood loss during operation period, length of stay at hospital in days, and perioperative complications, cervical Japanese Orthopaedic Association (C-JOA) score and visual analogue scale (VAS) pain score, Neck Disability Index (NDI), Nurick score, concomitant medication / treatment, new adverse events, radiographic data, revision surgery etc. will be collected. See Fig. 1 for more details.

In case severe adverse events occur, the subject can drop out at any time during the study.

### Outcome measures

#### Primary efficacy outcome

The primary efficacy endpoint is the C-JOA recovery rate at 12 months post operation, which is to be derived by Hirabayashi’s method [20] from original C-JOA scores. A set of C-JOA score per subject visit involves four aspects: upper limb motor function (range, 0 to 4 points), lower limb motor function (range, 0 to 4 points), sensory function (range, 0 to 6 points), and bladder function (range, 0 to 3 points). The total C-JOA score ranged from 0 (worst) to 17 (normal condition), with a lower score indicating more significant dysfunction. The recovery rate of C-JOA is calculated by the following Hirabayashi’s formula [20]:

$$ \mathrm{C}-\mathrm{JOA}\mathrm{recovery}\ \mathrm{rate}=\frac{\mathrm{Post}\ \mathrm{minus}\ \mathrm{Preoperative}\ \mathrm{C}-\mathrm{JOA}\ \mathrm{score}}{17\ \mathrm{minus}\ \mathrm{Preoperative}\ \mathrm{C}-\mathrm{JOA}\ \mathrm{score}}\times 100\% $$

#### Secondary outcomes

The secondary efficacy outcomes in this study included VAS pain score (range, 0 [no pain] to 10 [most severe]) [21], 10-item NDI (a total range of 0 to 50 points, the highest index the worst) [22] and 6-level Nurick disability grade (range, 0 [mild] to 5 [severe]) [23].

Safety endpoints.

The safety endpoints include any adverse events (AEs), perioperative complications, and adverse events of special interest. The AEs profile of both treatments will be evaluated by examining the incidence of AEs according to the National Cancer Institute Common Terminology Criteria for Adverse Events (NCI CTCAE, Version5.0) [24].

### Sample size calculation

We used SAS® software, V.9.4 (SAS Institute, North Carolina, USA) to estimate sample size. According to the principle of efficacy superiority design, the significance level α was set as 0.05 at two-sided. The primary efficacy endpoint of this study is the recovery rate of C-JOA at the 12th month of follow-up post operation. Assume that in severe COPLL patient population, the mean recovery rate of C-JOA in the ACAF group and the LAMP group at 12 months after operation is 63 and 51% (a mean difference of 12%), respectively, and the common standard deviation is 20%. When the two groups are randomly assigned 148 subjects in a 1:1 ratio, the statistical power can reach as high as 95%. If the subject dropout rate is 10% or less, 164 eligible subjects with severe COPLL should be randomly enrolled.

### Statistical analysis

The study includes three analysis populations per analysis plan. For the baseline and efficacy data, we will use full analysis set (FAS), which includes all subjects who were randomized into the study groups and received the study operation scheme. According to the principle of intention to treat, subjects in FAS would be analyzed using their randomly assigned group, regardless of the actual surgical treatment received. Per-protocol population, e.g. a subset of FAS will be established further for subjects with no major protocol deviation(s), which will be reviewed and determined by the sponsor before the study database is locked. Safety set (SS) is defined to include all subjects who have received the treatment of the study-specified operation (regardless of whether they participate in the randomized assignment or not) and will be the primary analysis population for safety data. The subjects in SS will be grouped according to the actual surgical treatment received.

In this study, a two-sided *p*-value of 0.05 or less (equivalently one-sided p-value of 0.025) will be considered to indicate significance for any statistical tests, unless otherwise specified in the interim analysis section. Software R, V.4.0.4 [25] and SAS®, V.9.4 will be used for statistical analysis. Such data as demographics, baseline characteristics, surgery operation, radiographic data and safety will be summarized according to treatment group.

The primary endpoint of this study is the C-JOA recovery rate at 12 months post operation. It will be analyzed with the use of analysis of covariance (ANCOVA), with random stratification factors (if appropriate) and the treatment group as fixed factors, and the baseline level of C-JOA score as covariate. The least squares mean (LSM), the difference from the control group and the confidence interval (CI) of each treatment group will also provided for statistical analysis.

In addition, a mixed model repeated measures (MMRM) will be used as supportive analysis when appropriate. MMRM analysis includes treatment group, time and time treatment group interaction as fixed effects, baseline level as covariate, and patients as random effects. When there are missing data, multiple imputation method will be applied as the primary analysis; those results from the last observation carry forward method (LOCF) and the as-observed data analysis will play roles as sensitivity analyses. If the data distribution does not meet the statistical hypothesis of ANCOVA, a set of rank-based analysis will be the primary one. The LSM, the difference from the control group and the CI of each treatment group will be also provided for statistical analysis. For MMRM and ANCOVA, the two-sided *p*-values will be provided when compared with the control group.

To investigate the consistency of the trial conclusions among different subpopulations, the subgroup analysis with regards to the primary efficacy endpoint will include but not limited to the following baseline factors: gender; age group; duration of complaining symptoms; diabetes status; smoking status; count of vertebrae segments involved; type of COPLL; K-line status; occupation ratio; C-JOA score; Nurick grade and status of hypersignal of spinal cord. The occupation ratio (OR) is defined as the biggest ratio of COPLL thickness to anteroposterior diameter of the bony spinal canal on the axial computed tomography image. A forest plot will be also provided as a useful display of estimated treatment effects across subgroups.

For VAS pain score and NDI index as continuous variables, the analysis method of between-group comparison is similar to those used for the primary endpoint. For cervical Nurick grades as an ordinal variable, the common odds ratio will be estimated by using ordinal logistic regression analysis (proportional odds model) to reflect the shift of full distribution of each grade between the two groups 12 months after operation. The adjusted and unadjusted common odds ratio and its 95% CIs will be calculated. When using the model to adjust prognostic factors, the important prognostic factors mainly include random stratification factor as appropriate, gender, age and baseline Nurick score, which will be clearly specified in the statistical analysis plan before the database lock.

For purposes indicative of statistical significance, we will use the Cochran Mantel Haenszel (CMH) method according to random stratification factors as appropriate to test the statistical hypothesis for important categorical adverse events. The incidences in each group and 95% CIs of incidence difference between the two groups will be calculated by Newcombe method [26]. Similar statistical analysis will also be conducted for each single adverse event of special interest, or Fisher’s exact test will be used for comparison between groups when applicable.

Radiographic data based on central review as their primary analyses will be compared between groups. When appropriate, we will also conduct exploratory analysis on the relationship between these imaging data and clinical outcomes, with an effort to predict the long-term clinical outcomes and complications with the use of preoperative and recent postoperative imaging data as well as other subject-level baseline characteristics. The statistical methods used for these exploratory analyses will be depicted in more details in the statistical analysis plan as appropriate.

### Interim analysis

Two formal interim analyses of efficacy data are planned when one-third and two-thirds of the enrolled subjects have completed at least 12 months of follow-up, respectively. The treatment efficacy and the occurrence of adverse events will be then reviewed by an independent Data and Safety Monitoring Board (DSMB), when the sample size might be also re-estimated and adjusted based on accumulative data of primary efficacy endpoint as necessary (Cui L et al., 1999) [27].

In the interim analysis of efficacy, the Haybitt-Peto boundary will be used (Haybitt 1971 [28]; Peto et al. 1976 [29]) to control the overall type I error. The alpha boundary is to be consumed at 0.0001 (one-sided and nominal level) at each interim analysis. The boundary value at the final stage will be kept at 0.025 at one-sided after the two scheduled interim analyses or be derived to maintain the Type I and Type II error probability levels if any ad hoc interim analysis is added.

### Study committees

Four committees are planned in the whole study. A Steering Committee was well organized to oversee the governance of the study. From independent external oversight, a DSMB was established and chaired by a surgical expert and also included another DSMB statistician and one radiologist as well. The members of the DSMB serve in an individual capacity and provide their expertise and recommendations on a timely basis to the study Steering Committee.

A Technical Training Committee (TTC) was organized to develop a training manual on ACAF technique, carry out field lecture at each branch centre and perform demo surgery as appropriate. Besides, all of the radiographic scans (X-ray, CT and magnetic resonance imaging [MRI]) in the study will be forwarded to an independent Radiographic review Committee (IRC) for retrospective review on a regular basis.

## Discussion

In the three recent exploratory studies [13, 14, 16], the pilot use of ACAF technique (or called vertebral body sliding osteotomy [VBSO] in principle) showed promising treatment benefits in COPLL patients. The novel surgery technique can achieve anterior direct decompression without separating and removing ossification. Its basic principle is to expand the spinal canal by anteriorly translating the involved vertebral bodies with any ossified masses [15, 16] (Fig. 2). Based on surgeons’ preference, this novel technique was initially proposed more suitable for severe COPPL patients as opposed to continue conventional anterior or posterior approaches for non-severe patients. Patients included in the small-sized studies are those with OPLL involved three or more vertebrae and a canal occupying ratio of more than 60% [13], or those with at least three levels of cervical compression caused by OPLL [14], or those with negative K-line and a large OPLL mass or kyphotic cervical alignment [16]. In line with these findings, we designed this new study to have a statistical hypothesis enriched in the severe COPLL subpopulation. To make a balanced assignment of ACAF versus LAMP across each stratum of patients and validity of subsequent statistical comparison, therefore, we pre-specified several stratification factors when conducting randomized assignment of the two treatments among enrolled subjects. This had better help set up any statistical modeling in the data analysis.

The use of anterior controllable antedisplacement and fusion belongs to one of anterior approaches, which are usually high technically demanding. However, the excellent neurologic outcome noted in the severe COPPL patients might be extended to the whole COPPL population as long as the spine surgeons in the study are rich in spine surgery experience and can also follow a standardized operation schema after uniform training held by TTC. This is why our study operation team in the sponsor’s site will design a training manual of ACAF technique, carry out field lecture and perform surgery in concert for the first several cases of each site. On the other hand, the study site is included as one of randomization factors, which provides a data basis for analyzing potential site effect in the future.

Furthermore, for purpose of better data quality, the study team will employ a dedicated clinical monitor team for source data verification. The radiographic data will be centrally reviewed by the IRC. Due to the limited OPLL patients at some of our study sites, it may take a long time to recruit the sample size required for this study. Moreover, we face the challenge of subjects’ follow-up issue to collect primary efficacy data especially in the long run. We will also utilize additional patient accrual and follow-up services from a dedicated third-party clinical research coordinator team. An electronic data capture system with audit trails will also be used in the study.

Based on this adequate and well-controlled study, we expect to obtain solid study data and with them to demonstrate whether this novel ACAF technique is more favorable than LAMP in the selected study population. We have a plan to formulate an expert consensus with our study findings and also develop a COPLL-specific clinical practice guideline as appropriate. All of these after publication will be shared with participating hospitals and the academic communities to promote the clinical management of severe COPLL patients.

## Data Availability

Not Applicable.

## Abbreviations

ACAF: Anterior controllable antedisplacement and fusion
ANCOVA: Analysis of covariance
C-JOA: Cervical- Japanese Orthopaedic Association
COPLL: Cervical ossification of the posterior longitudinal ligament
LAMP: Posterior laminoplasty
NDI: Neck disability index
VAS: Visual analogue scale
ACAF: Anterior controllable antedisplacement and fusion
ANCOVA: Analysis of covariance
CI: Confidence interval
C-JOA: Cervical- Japanese Orthopaedic Association
CMH: Cochran Mantel Haenszel
CONSORT: Consolidated Standards of Reporting Trials
COPLL: Cervical ossification of the posterior longitudinal ligament
CT: Computed tomography
DSMB: Data and Safety Monitoring Board
FAS: Full analysis set
IRC: Independent Radiographic review Committee
IEC: Independent ethics committee
LAMP: Posterior laminoplasty
LOCF: Last observation carry forward
LSM: Least squares mean
MMRM: Mixed model repeated measures
MRI: Magnetic resonance imaging
NCI CTCAE: National Cancer Institute Common Terminology Criteria for Adverse Events
NDI: Neck disability index
OPLL: Ossification of the posterior longitudinal ligament
SS: Safety set
TTC: Technical Training Committee
VAS: Visual analogue scale
VBSO: Vertebral body sliding osteotomy
